# Supplementary material for: Mechanisms governing the pioneering and redistribution capabilities of the non-classical pioneer PU.1
Source: Nat Commun. 2020 Jan 21;11:402. doi: 10.1038/s41467-019-13960-2 (PMC6972792; doi:10.1038/s41467-019-13960-2)
Supplement: Supplementary file 7 — Source data [file 41467_2019_13960_MOESM7_ESM.zip › Source_Data/FigureS2/FigureS2B_MotifScanOutput/KmeansCluster1/homerResults/motif1.similar.html]

motif1

## Information for motif1

A
T
G
C
C
G
T
A
A
T
G
C
A
C
G
T
A
C
G
T
A
G
T
C
A
G
T
C
A
G
C
T
A
G
T
C
G
C
A
T
G
C
A
T
G
C
A
T
G
A
C
T
A
G
C
T
  
Reverse Opposite:  

T
C
G
A
C
T
G
A
C
G
T
A
C
G
T
A
C
G
T
A
T
C
A
G
T
C
G
A
C
T
A
G
A
C
T
G
C
G
T
A
C
G
T
A
T
A
C
G
G
C
A
T
A
T
C
G
  

|  |  |
| --- | --- |
| p-value: | 1e-3599 |
| log p-value: | -8.289e+03 |
| Information Content per bp: | 1.640 |
| Number of Target Sequences with motif | 4251.0 |
| Percentage of Target Sequences with motif | 83.50% |
| Number of Background Sequences with motif | 1668.1 |
| Percentage of Background Sequences with motif | 4.21% |
| Average Position of motif in Targets | 101.6 +/- 19.4bp |
| Average Position of motif in Background | 98.1 +/- 73.2bp |
| Strand Bias (log2 ratio + to - strand density) | 0.1 |
| Multiplicity (# of sites on avg that occur together) | 1.05 |
| Motif File: | file (matrix) reverse opposite |

### Similar de novo motifs found

|  |  |  |  |  |  |  |  |
| --- | --- | --- | --- | --- | --- | --- | --- |
| Rank | Match Score | Redundant Motif | P-value | log P-value | % of Targets | % of Background | Motif file |
| 1 | 0.965 | T C G A C T G A C T G A C G T A T C A G T C G A C T A G A C T G C G T A C G T A T A C G C G A T T A C G | 1e-3364 | -7746.634711 | 80.79% | 4.35% | motif file (matrix) |
| 2 | 0.925 | A T G C G C T A A T G C A C G T A C G T A G T C A G T C A G C T A T G C G C A T | 1e-2735 | -6298.700097 | 80.38% | 7.93% | motif file (matrix) |
| 3 | 0.918 | A T G C G C T A A T G C A C G T A C G T A G T C A G T C A G C T A G T C | 1e-2543 | -5857.767559 | 86.33% | 12.97% | motif file (matrix) |
| 4 | 0.922 | C T G A C G T A T C A G T C G A T C A G A T C G C T G A C T G A T A C G C G A T T A C G | 1e-2499 | -5756.274518 | 89.31% | 15.60% | motif file (matrix) |
| 5 | 0.907 | C T G A C G T A T A C G C T G A C T A G C T A G T C G A C T G A T A C G C G A T T A C G T C A G | 1e-2345 | -5400.808599 | 77.16% | 9.34% | motif file (matrix) |
| 6 | 0.963 | T A G C G T C A A T G C A C G T A C G T A G T C A G T C G A C T G A T C G C T A G C A T G C A T | 1e-2303 | -5303.805902 | 59.95% | 2.91% | motif file (matrix) |
| 7 | 0.907 | A T G C C G T A A G T C A C G T A C G T A G T C A G T C A G C T | 1e-2126 | -4897.383394 | 73.66% | 9.42% | motif file (matrix) |
| 8 | 0.890 | A T G C C G T A A G T C A C G T A C G T A G T C A G T C | 1e-2004 | -4616.026136 | 68.85% | 8.11% | motif file (matrix) |
| 9 | 0.894 | C T G A T C G A C T G A T C A G C T G A C T A G C T A G G C T A C G T A T C A G G C A T T C G A C T G A | 1e-1976 | -4551.684648 | 81.54% | 15.77% | motif file (matrix) |
| 10 | 0.837 | C T G A T C G A C T G A C A T G C T G A C T A G A C T G C G T A C G T A T A C G | 1e-1894 | -4363.386646 | 72.70% | 11.09% | motif file (matrix) |
| 11 | 0.811 | C G T A A T G C A C G T A C G T A G T C A G T C A T C G A G T C | 1e-1869 | -4305.069235 | 71.79% | 10.85% | motif file (matrix) |
| 12 | 0.887 | A C T G C T A G T C A G C T G A A C T G C T A G C G T A C G T A A C T G A G C T T C A G T C G A | 1e-1820 | -4190.820888 | 59.10% | 5.44% | motif file (matrix) |
| 13 | 0.839 | C T G A G C T A C G T A G C T A T A C G T C G A C T A G A C T G G T C A C G T A T A C G | 1e-1587 | -3655.010987 | 67.59% | 11.52% | motif file (matrix) |
| 14 | 0.934 | C G A T A C G T C G A T C T A G C T G A A C T G A C T G C G T A C G T A A T C G G C A T T A C G T A G C | 1e-1490 | -3433.006390 | 39.89% | 1.59% | motif file (matrix) |
| 15 | 0.877 | C T G A A T G C G C A T C G A T A G T C G T A C C G A T A T G C C G A T G C A T A G C T A G C T | 1e-1338 | -3081.234088 | 36.87% | 1.58% | motif file (matrix) |
| 16 | 0.809 | A G T C A C G T A C G T A G T C A G T C A G C T A G T C | 1e-1130 | -2602.114004 | 66.10% | 16.77% | motif file (matrix) |
| 17 | 0.777 | A T G C A G C T A C G T A G T C A G T C A G C T A G T C | 1e-1008 | -2321.574854 | 53.31% | 10.75% | motif file (matrix) |
| 18 | 0.761 | A G T C A C G T A C G T A G T C A G T C A G T C A G T C A G C T | 1e-867 | -1997.678180 | 54.41% | 13.50% | motif file (matrix) |
| 19 | 0.791 | G T A C A C G T A C G T T A G C A G T C A C G T A G T C G C A T A G T C | 1e-818 | -1883.720966 | 56.59% | 15.76% | motif file (matrix) |
| 20 | 0.833 | C G T A G A C T T C G A C T G A C T A G A C T G G T C A T G C A T A C G G A C T A T C G | 1e-706 | -1627.012135 | 21.80% | 1.16% | motif file (matrix) |
| 21 | 0.775 | C T G A A T G C A C G T A C G T G T A C G A T C G A T C G A C T G C T A G C A T | 1e-612 | -1410.425590 | 26.40% | 3.19% | motif file (matrix) |
| 22 | 0.772 | A C G T A C T G A G T C G A C T A C G T A G T C G A T C G A C T A T G C G C A T A C G T A C G T A G C T G A T C | 1e-541 | -1247.402530 | 24.69% | 3.24% | motif file (matrix) |
| 23 | 0.854 | C T G A A C T G T C A G A T C G G C T A G T C A A T C G A G C T T A C G | 1e-451 | -1039.546963 | 22.18% | 3.22% | motif file (matrix) |
| 24 | 0.792 | C T A G T G A C C G T A A G T C A C G T C A G T A G T C A T G C | 1e-431 | -992.758100 | 25.30% | 4.78% | motif file (matrix) |
| 25 | 0.843 | C G T A C T A G A C G T T A C G C G T A C T A G T C A G G T C A C G T A T C A G A C G T C T A G T C A G | 1e-415 | -956.066894 | 20.64% | 3.02% | motif file (matrix) |
| 26 | 0.760 | C G T A G T A C A C G T A C G T G T A C G T A C G A T C C G A T C T A G A C G T A C G T G T A C | 1e-245 | -565.732043 | 9.37% | 0.79% | motif file (matrix) |
| 27 | 0.758 | T G C A C G T A C G T A A C T G A T G C C T G A A C T G C T G A C G T A T A C G C A G T A T C G T G C A | 1e-143 | -330.471001 | 4.83% | 0.29% | motif file (matrix) |
